# *Uncaria tomentosa* extract exerts antimicrobial activity against boar seminal bacteria and influences sperm resilience under different conditions

**DOI:** 10.3389/fvets.2025.1558650

**Published:** 2025-03-21

**Authors:** Maria Scaringi, Eliana Pintus, Pavel Nový, Katerina Božiková, Petr Maršík, Jose Luis Ros-Santaella

**Affiliations:** ^1^Department of Veterinary Sciences, Faculty of Agrobiology, Food and Natural Resources, Czech University of Life Sciences Prague, Prague, Czechia; ^2^Department of Food Science, Faculty of Agrobiology, Food and Natural Resources, Czech University of Life Sciences Prague, Prague, Czechia

**Keywords:** antibiotics, antioxidant, cat's claw, oxidative stress, pig, plant extract, secondary metabolites, semen storage

## Abstract

*Uncaria tomentosa* (UT) or cat's claw, is a vine belonging to the Rubiaceae family and native to South and Central America. Various parts of the plant, including bark, showed many therapeutic activities (e.g., antioxidant and antibacterial), but the *in vitro* effects on gametes have still not been investigated. During boar semen storage for artificial insemination purposes, oxidative stress and bacterial contamination negatively affect sperm quality. In this study, we evaluated the tolerance of boar sperm to UT ethanolic extract at four concentrations (1.6 to 0.025 μg/mL). The analyses were carried out on sperm samples under oxidative stress, induced by H_2_O_2_ and Fe^2+^/Ascorbate, and during 96 h of semen storage at 17°C. The antibacterial activity of the extract (1,024 to 8 μg/mL) was tested against commercial strains and bacteria isolated from the semen. The treatments ranging from 0.4 to 0.025 μg/mL protected sperm membrane (*p* < 0.05) and preserved some kinetic parameters in samples under oxidative stress (Fe^2+^/Ascorbate). During semen storage, the extract did not show any cytotoxicity, and mean values of some sperm parameters were higher than the control group, although not significant (*p* > 0.05). All tested Gram-positive bacteria exhibited growth inhibition. The most frequently isolated Gram-negative bacteria from semen (i.e., *Citrobacter koseri, Pseudomonas aeruginosa, Stenotrophomonas maltophilia*) also showed complete growth inhibition, while the remaining strains showed a partial decrease in growth. Taken together, our findings show that *Uncaria tomentosa* is a promising plant-based additive for boar semen storage.

## 1 Introduction

*Uncaria tomentosa* (UT), commonly known as cat's claw, is a climbing vine native to the tropical rainforests of South and Central America. Belonging to the Rubiaceae family, it is characterized by distinctive claw-shaped thorns at the leaf junctions, hence its common name (1). Indigenous communities, such as the Asháninka people of Peru, widely use UT even alluding to miraculous healings (2). Traditionally, various parts of this vine, particularly the bark and leaves, have been employed in medicinal preparations to treat a wide range of ailments, including asthma, menstrual irregularities, fever, infections, mental health conditions like anxiety, and wound healing. It has also been used as a contraceptive and to address conditions like hemorrhages and weakness (1–3). The *Uncaria tomentosa* can be categorized into “pentacyclic alkaloid-type” or “tetracyclic alkaloid-type” based on the dominant group of oxindole alkaloids present. These distinct chemical profiles correlate with varying pharmacological and biological properties (4, 5). The pharmacological aspects of this plant include antimicrobial, antiprotozoal, anti-neoplastic, cardiovascular, anti-inflammatory, and antioxidant activities. All the properties attributed to the plant are due to the presence of secondary metabolites; among them, the antioxidant and the antibacterial activities are attributed to the presence of polyphenols and alkaloids (5–8). Previous research has reported cat's claw's antioxidant properties in various cell types (9, 10) and antibacterial activity against both Gram-positive (Gram+) and Gram-negative (Gram–) bacteria (11, 12). However, its effects on sperm cells and seminal bacteria are still unknown. In recent years, there has been growing interest in utilizing natural products as additives for semen preservation. Plants extracts are being explored as potential replacements for traditional antioxidants and antibiotics, with the goal of improving overall semen quality (13).

Artificial insemination has become the predominant method for pig production globally over the past few decades. Its adoption has surged worldwide with the percentage of artificially inseminated sows ranging from 80 to 98% in several European countries (14). This trend is projected to continue over the next decade as increasing population and shifting dietary habits in developing countries drive greater demand for pork (15). One of the biggest advantages of artificial insemination is the possibility to choose the best boar ejaculates in terms of fertility, cutting out the infertile and subfertile samples. This careful selection is essential as a single ejaculate can fertilize up to 10–15 sows (16). Selected semen samples are typically stored at 15–20°C for 1 to 5 days in specialized extenders. These extenders increase semen volume to achieve the desired artificial insemination dose while prolonging sperm viability, thanks to the presence of nutrients and substances that help to maintain the functional characteristics of the spermatozoa (17). Sperm quality deteriorates during storage due primarily to oxidative stress and bacterial contamination (18, 19). To mitigate this, the extenders commonly include antioxidants and antibiotics to protect sperm cells from bacterial and oxidative damage (20, 21).

Because of the antioxidant and antimicrobial attributes previously reported of this plant and the fact that it has never been tested on animal gametes and bacteria isolated from semen, we aimed to: i) characterize the main secondary metabolites and the antioxidant capacity of the extract; ii) assess its potential protective effect on sperm cells under oxidative stress and during semen storage at 17°C up to 96 h; iii) determine its antibacterial spectrum against the most frequent bacteria isolated from boar semen and related commercial strains.

## 2 Materials and methods

All reagents were purchased from Merck (Darmstadt, Germany), unless otherwise indicated.

### 2.1 Preparation of *U. tomentosa* extract

*Uncaria tomentosa* bark, native to Peru, was purchased from a specialized herbal shop (OXALIS Retail s.r.o.). The stock extract was prepared by adding 20 mL of ethanol to 4 g of dried bark (20% mass/volume) and stirred at 400 rpm for 24 h. Afterwards, the extract was filtered with a paper filter (Whatman n. 4) and stored at −80°C.

### 2.2 Characterization of *U. tomentosa* extract

#### 2.2.1 Preparation of the dry extract and determination of extraction yield

The stock extract was dried by a nitrogen blowdown. The extraction yield (%) was determined on the dry extract as follows:


$$\begin{array}{l} extraction\;yield\;\left(\%\right)=\frac{weight\;of\;the\;dry\;extract}{weight\;of\;the\;Uncaria\;tomentosa\;bark}x100 \end{array}$$

Finally, the dry residue was solubilized in ethanol to obtain a new stock solution at 102.4 mg/mL.

#### 2.2.2 Total phenolic content (TPC)

The TPC was determined spectrophotometrically as previously described with minor modifications (22, 23). Firstly, 100 μL of cat's claw extract, gallic acid standard (32, 16, 8, 4, and 2 μg/mL) or distilled water (blank) were transferred to a 96-well microtiter plate. Then, 25 μL of Folin-Ciocalteu reagent (2N) was added to each well and the plate was placed in an orbital shaker at 50 rpm for 10 min. Seventy-five microliters of a 12% (w/v) sodium carbonate (Na_2_CO_3_) solution was added to initiate the reaction. The plate was incubated in the dark at 37°C for 1 h. Absorbance was measured at a wavelength of 700 nm on a Biotek Synergy H1M microplate reader (Agilent, Santa Clara, CA, USA). Results were expressed as gallic acid equivalents (mg GAE/g dry extract). The analysis was carried out five times.

#### 2.2.3 Total flavonoid content (TFC)

The TFC was quantified spectrophotometrically using a modification of the previously described aluminum chloride (AlCl_3_) method (24). The assay was conducted in a 96-well microtiter plate: 100 μL of cat's claw extract, quercetin standard (100, 50, 25, 12.5, 6.25, 3.12, 1.56, and 0.78 μg/mL) or distilled water (blank) were combined with AlCl_3_. The plate was incubated in the dark at room temperature for 1 h and the absorbance was measured at the wavelength of 420 nm on a Biotek Synergy H1M microplate reader (Agilent, Santa Clara, CA, USA). Results were expressed as quercetin equivalents (μg QE/g dry extract). The analysis was performed five times.

#### 2.2.4 ABTS decolorization assay

The antioxidant capacity of the extract was determined spectrophotometrically by the previously described ABTS [2,2′-azinobis-(3-ethylbenzothiazoline-6-sulfonic acid)] decolorization assay with some changes (25). Briefly, the ABTS radical cation (ABTS^•+^) was produced by adding 5.05 μL of ammonium persulfate (245 mM) to 500 μL of ABTS solution (7 mM). The solution was incubated overnight in the dark at room temperature and, before the experiment, it was diluted to 1% v/v in phosphate-buffered saline (PBS) solution. Ten microliters of cat's claw extract, Trolox standard (5, 2.5, 1.25, and 0.625 μg/mL) or PBS (blank) were added to 96-well microtiter plate. Afterwards, 190 μL of ABTS^•+^ solution was added to each well and the plate was incubated for 5 min in the dark at room temperature. The absorbance was measured at 734 nm on a Biotek Synergy H1M microplate reader (Agilent, Santa Clara, CA, USA). Results were expressed as Trolox equivalents (mmol TE/g dry extract). The analysis was run five times.

#### 2.2.5 DPPH radical scavenging assay

The antioxidant activity of the cat's claw extract was determined by slightly modifying the previously described DPPH (1,1-Diphenyl-2-picryl-hydrazyl) radical scavenging assay (26). An amount of 100 μL of DPPH solution (0.25 mM) dissolved in ethanol was added to 100 μL of the extract, Trolox standard (256, 128, 64, 32, 16, 8, 4, and 2 μg/mL) or ethanol (blank) into 96-well microtiter plate. The plate was incubated in the dark at room temperature for 30 min and the absorbance was measured at 517 nm on a Biotek Synergy H1M microplate reader (Agilent, Santa Clara, CA, USA). Results were expressed as half-minimal inhibitory concentrations (IC_50_ in μg/mL) and Trolox equivalents (mg TE/g dry extract). The analysis was carried out five times.

#### 2.2.6 Oxygen radical absorbance capacity (ORAC)

The ORAC was established by a previously developed method (27) with some changes. All reagents and cat's claw extract were prepared in phosphate buffer (75 mM, pH 7.0). A volume of 150 μL of fluorescein (48 nM) was added to 25 μL of cat's claw extract, Trolox standard (8, 4, 2, 1, and 0.5 μg/mL) or phosphate buffer (blank) in a black 96-well microtiter plate, then incubated in the dark for 10 min at 37°C. Afterwards, 25 μL of 2,2′-Azobis (2-methylpropionamidine) dihydrochloride (153 mM) was added in each well. Fluorescence changes were measured in 1-min intervals for 2 h with excitation and emission wavelengths set respectively at 487 nm and 528 nm on a Biotek Synergy H1M microplate reader (Agilent, Santa Clara, CA, USA). The total ORAC of the cat's claw was quantified as area under the curve as previously proposed by Cao et al. (28). Results were expressed as Trolox equivalents (mmol TE/g dry extract). The analysis was run five times.

#### 2.2.7 Ultra-high performance liquid chromatography/mass spectrometry (UHPLC/MS)

The analysis of UT ethanolic extract (10 μg/mL) was performed using UHPLC/MS with high resolution and high-accuracy mass (HRAM). The analytical system consists of an ultra-high-performance chromatograph Ultimate 3,000 (Thermo Fischer Scientific, Waltham, MA, USA) with an HRAM mass Q-TOF spectrometer Impact II (Bruker Daltonik, Bremen, Germany). A reversed phase column Acclaim RSLC 120 C18 (2.2 μm, 2.1 × 100 mm, Thermo-Fischer Scientific, USA) tempered at 35°C was used for separation. Gradient elution with mobile phase consisting of formic acid in water (0.2% v/v; solvent A) and methanol (solvent B) with flow rate of 250 μg/min started at 2% B (0–1 min), then increased to 100% B in 25 min, where it was kept for 10 min until 35 min and followed by equilibration at initial conditions from 37 to 47 min. The sample injection volume was 5 μL. MS analysis was performed using ESI ionization in positive mode. Data were collected in full-scan detection mode with resolution of 60,000, in mass range from 80 to 1,200 Da and sampling frequency of 1 Hz. Data acquisition and processing was carried out by otofControl 4.0, HyStar 3.2 and DataAnalysis 4.3 software (all Bruker Daltonik, Bremen, Germany). The relative amount of the alkaloids was expressed as the peak area of extracted chromatogram at the corresponding m/z with accuracy of ±0.002.

### 2.3 Semen collection and processing

Semen doses from 14 Duroc boars were purchased from a pig-breeding company (Lipra Pork, a.s., Czech Republic). Semen was collected by the gloved hand method and diluted 1:1 with the short-term extender Beltsville Thawing Solution (BTS, D-glucose 37 g/L, sodium citrate 6 g/L, ethylenediaminetetraacetic acid 1.25 g/L, sodium bicarbonate 1.25 g/L, potassium chloride 0.75 g/L, gentamicin 0.25 g/L). The samples were transported to the laboratory at 25°C. In the laboratory, the ejaculates were again diluted 1:1 with BTS and centrifuged (167 g, 17°C, 3 min) to remove abnormal cells and debris; the supernatants were transferred to new tubes (29). An aliquot of each ejaculate was fixed with 0.3% formaldehyde in PBS (v/v) to evaluate sperm morphology. The samples were analyzed under a phase contrast microscope (Nikon Eclipse E200, Nikon, Japan) at 40× objective, evaluating 200 cells and grouping them in one of the six categories: normal sperm cells, presence of proximal droplets, presence of distal droplets, head abnormalities, flagellum abnormalities, and other abnormalities. To minimize the impact of individual boar variability, semen pools from two to four boars were created for each experimental session. Each pool had a minimum of 75% sperm cells with normal morphology. An aliquot of the pool was added to 0.3% formaldehyde solution to assess the sperm concentration using a Bürker chamber. The semen was diluted to 20 × 10^6^ sperm cells/mL in BTS. For each experiment, the semen pool was split into five tubes, the control (Ctr) tube was supplemented with ethanol (final concentration 0.1% v/v); the remaining tubes were supplemented with different concentrations of UT extract, selected based on preliminary tests. The final concentrations of the extract in the semen samples were 1.6 μg/mL (UT 1.6), 0.4 μg/mL (UT 0.4), 0.1 μg/mL (UT 0.1), and 0.025 μg/mL (UT 0.025). Each experiment was replicated six times using six different semen pools.

#### 2.3.1 Experiment 1: sperm samples under oxidative stress

The effects of the extract on semen were evaluated under oxidative stress induced by two reactive oxygen species (ROS)-generating systems [Fe^2+^/Ascorbate (Fe^2+^/Asc) and H_2_O_2_], as previously reported (29, 30). In the first experimental group, oxidative stress was induced by adding H_2_O_2_ (final concentration 10 μM) to the control group with induced oxidative stress (Ctr-Ox) and UT treatments. In the second experimental group, oxidative stress was induced by Fe^2+^/Asc, by the addition of a solution containing iron (II) sulfate (FeSO_4_; final concentration 0.05 mM) and sodium ascorbate (final concentration 0.5 mM) to Ctr-Ox and UT treatments. For each experimental group, a control group without oxidative stress (Ctr) was included. The analyses were performed after incubating the samples at 38°C in a water bath for 1.5 h under the H_2_O_2_ system and for 3 h under the Fe^2+^/Asc system.

#### 2.3.2 Experiment 2: sperm samples stored at 17°C

Semen samples were stored in well-closed tubes within an air-conditioned box for boar semen liquid storage. This environment provided consistent air circulation and maintained a constant temperature of 17°C. The sperm analyses of Ctr and UT treatments were performed after 48 h and 96 h of semen storage. Before running the analyses, the samples were incubated at 38°C for 20 min in a water bath.

#### 2.3.3 Sperm motility and kinetics

Sperm motility and kinetics were evaluated using a Computer Assisted Sperm Analyzer (NIS-Elements; Nikon, Tokyo, Japan, and Laboratory Imaging, Prague, Czech Republic) as previously described (30). After loading 2 μL of sperm sample into a pre-warmed (38°C) Leja chamber (Leja Products BV, The Netherlands, chamber depth: 20 μm), at least 400 cells were analyzed for each sample in six random fields. The parameters evaluated were: total motility (TM, %), progressive motility (PM, %), average path velocity (VAP, μm/s), curvilinear velocity (VCL, μm/s), straight-line velocity (VSL, μm/s), amplitude of lateral head displacement (ALH, μm), beat-cross frequency (BCF, Hz), linearity (LIN=VSL/VCL, %), and straightness (STR=VSL/VAP, %). The standard CASA settings were as follows: frames per second, 60; minimum of frames acquired, 31; number of fields analyzed, 6; VAP ≥ 10 μm/s to classify a spermatozoon as motile; STR ≥ 80% to classify a spermatozoon as progressive. All videos were visually inspected to remove abnormal sperm pathways and non-sperm cells.

#### 2.3.4 Sperm plasma membrane integrity

**Figure 1:**
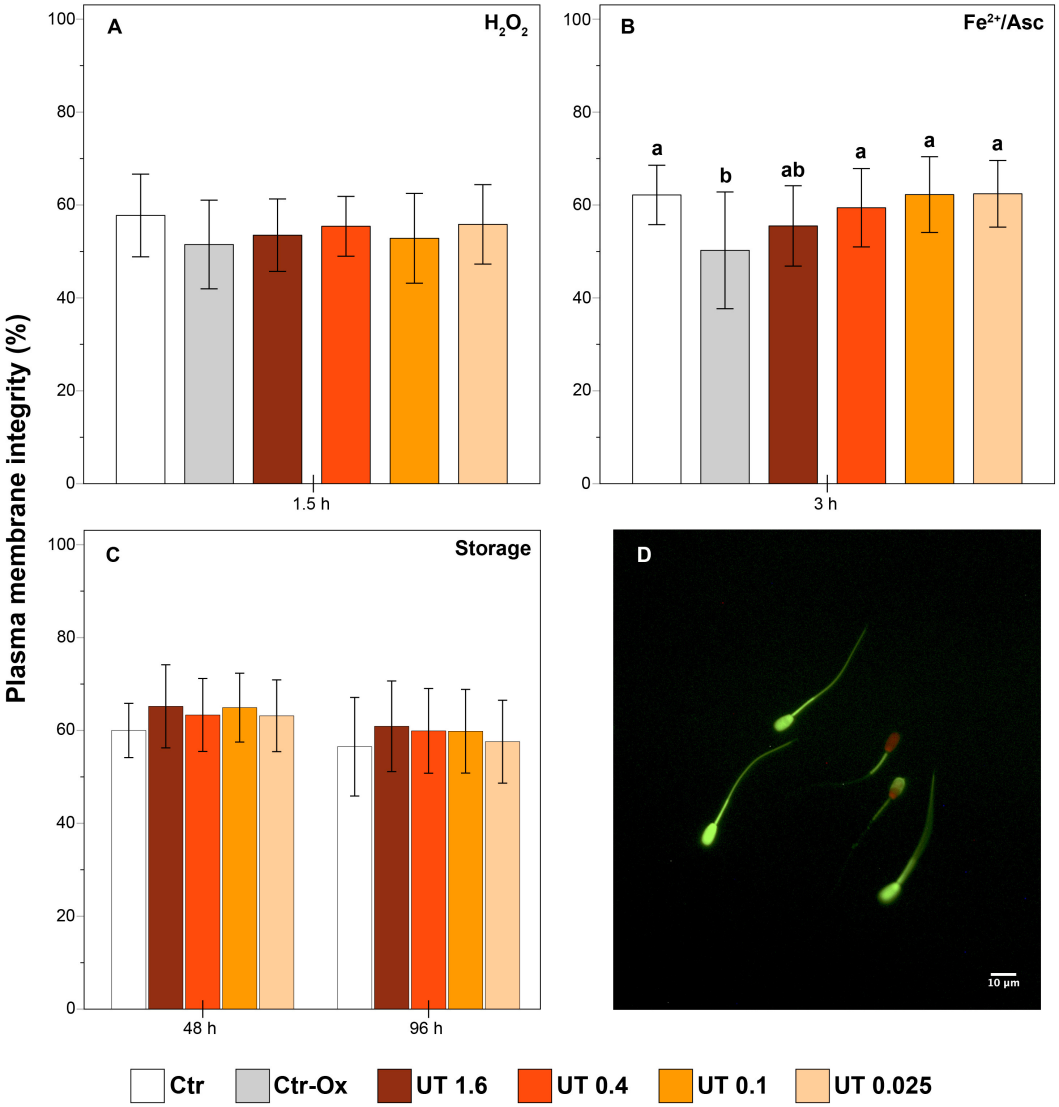
Effect of *Uncaria tomentosa* extract on plasma membrane integrity **(A)** effect under oxidative stress induced by H_2_O_2_; **(B)** effect under oxidative stress induced by Fe^2+^/Ascorbate; **(C)** effect during storage at 17°C; **(D)** sperm cells with intact plasma membrane (totally green) and damaged plasma membrane (partially or totally red) under epi-fluorescence microscopy. Different letters indicate significant differences (*p* < 0.05) between treatments. Treatments: Ctr, control; Ctr-Ox, control with induced oxidative stress; UT 1.6, *Uncaria tomentosa* 1.6 μg/mL; UT 0.4, *Uncaria tomentosa* 0.4 μg/mL; UT 0.1, *Uncaria tomentosa* 0.1 μg/mL; UT 0.025, *Uncaria tomentosa* 0.025 μg/mL. The data are shown as the mean ± standard deviation of six replicates.

Sperm plasma membrane integrity was assessed as previously described with minor modifications (31). An aliquot of 20 μL of semen sample was mixed with 80 μL of staining solution, which contained 75 μL of PBS, 2 μL of propidium iodide (stock solution: 0.5 mg/mL in PBS), 2 μL of carboxyfluorescein diacetate (stock solution: 0.46 mg/mL in dimethyl sulfoxide, DMSO), and 1 μL of formaldehyde 0.3% in PBS. The samples were incubated for 10 min at 38°C in the dark. Then, for each sample, 200 spermatozoa were evaluated under epi-fluorescence microscopy (Nikon Eclipse E600, Nikon, Japan; 40× objective). Sperm cells with intact plasma membranes show only green fluorescence over the head region (Figure 1D).

### 2.4 Antimicrobial assays

#### 2.4.1 Bacteria isolated from boar semen

Bacteria were isolated from 38 raw ejaculates obtained from 26 animals through cultivation on universal (Plate count agar, Blood agar) as well as on selective cultivation media (MacConkey agar, *Pseudomonas* isolation agar, Manitol salt agar, TBX agar), all obtained from Oxoid (Basingstoke, UK). Sample aliquots of 50 μL were plated on agar plates using a spiral plate inoculator EasySpiral (Interscience, Saint Nom, France) and incubated at 37°C for 24–48 h. Colonies with different appearance were passaged one or more times in order to obtain isolated colonies. Freshly grown colonies were identified using the standard procedure described by Bruker Daltonics for the AutoFlex Speed MALDI-TOF MS identification (ethanol-formic acid extraction procedure and then mixed with HCCA matrix) using FlexControl 3.4; MALDI Biotyper Compass version 4.1; and flexAnalysis version 3.4 software (Bruker Daltonics, Bremen, Germany). The isolates were stored at −80°C until the evaluation of the extract's minimum inhibitory concentration (MIC) and its effect on bacterial kinetic growth.

*Uncaria tomentosa* extract was tested on 12 bacterial strains: five Gram+ and seven Gram–. The standard bacterial strains from American Type Culture Collection (ATCC) were purchased from Oxoid (Basingstoke, UK), while other strains were the most frequently isolated from porcine semen samples (IS, Isolated from Semen), in this and our previous study (32). The Gram+ bacteria tested were: *Enterococcus faecalis* (ATCC 29212 and IS 21751), *Staphylococcus aureus* (ATCC 29213), *Staphylococcus epidermidis* (IS 2152), and *Staphylococcus pasteuri* (IS 22062). The Gram– bacteria tested were: *Citrobacter koseri* (IS 2111), *Escherichia coli* (ATCC 25922 and IS 21710), *Klebsiella aerogenes* (IS 2137), *Pseudomonas aeruginosa* (ATCC 27853 and IS 2216), and *Stenotrophomonas maltophilia* (IS 2107). Although *E. coli* was not frequently isolated from semen in our studies, it was included due to its documented detrimental effects on sperm cells. Several studies have shown that *E. coli* can reduce sperm motility, damage the acrosome (33) and cause sperm agglutination, which negatively impact litter size (34).

#### 2.4.2 MIC and bacterial kinetic growth

The MIC and the bacterial kinetic growth were determined by the broth microdilution method in 96-well microtiter plates following the guidelines of Clinical and Laboratory Standards Institute (35). The stock solution of *U. tomentosa* 102.4 mg/mL was diluted 100 times in Mueller-Hinton broth (MHB; Oxoid, Basingstoke, UK) to obtain the starting concentration; afterwards, serial dilutions in MHB were carried out directly in the wells (1,024, 512, 256, 128, 64, 32, 16, and 8 μg/mL). Gentamicin was used as positive control (64, 32, 16, 8, 4, 2, 1, 0.5, 0.25, and 0.125 μg/mL). Negative and sterile controls were included in the analysis. A bacterial suspension from each strain was incubated overnight at 37°C in MHB. On the day of the experiment, the overnight culture was standardized to 0.5 McFarland and then added to the wells (except for sterile control) to reach the final concentration of 5 × 10^4^ CFU/mL. The inoculum concentration was established based on the maximum contamination rates commonly observed in boar semen (33). The MICs were evaluated by the unaided eye after 24 h of incubation at 37°C and expressed as μg/mL of dry extract. Growth kinetics of the bacteria that showed a MIC ≥ 1,024 μg/mL were monitored at 1-h intervals over a 24 h period. Optical density measurements were taken at the wavelength of 512 nm and the temperature of 37°C using a Biotek Synergy H1M microplate reader (Agilent, Santa Clara, CA, USA). The readings were then used to generate kinetic growth curves. All tests were carried out at least in duplicate.

### 2.5 Statistical analyses

Data were analyzed using the SPSS 29 statistical software package (IBM Inc, Chicago, IL, USA). A generalized linear model was used to analyze the effects of treatment on the sperm variables on semen samples under oxidative stress and to analyze the effects of time and treatment on the sperm variables during semen storage at 17°C. All results are expressed as mean ± standard deviation (SD). The statistical significance was set at *p* < 0.05.

## 3 Results

### 3.1 Characterization of *U. tomentosa* extract

**Figure 2:**
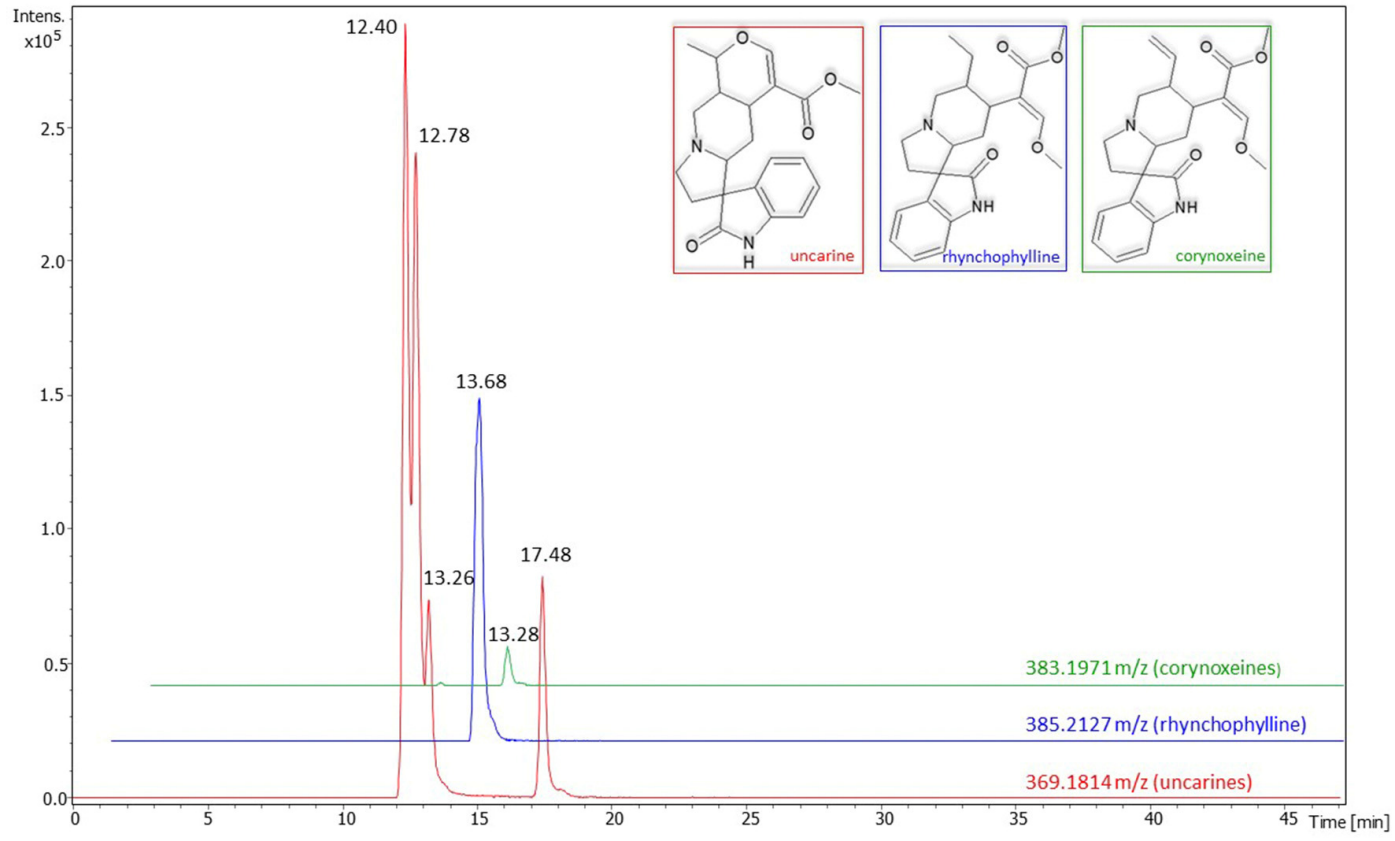
Chromatograms of alkaloids detected in *Uncaria tomentosa* extract. The numbers on the peaks represent the retention times. Intens., intensity; m/z, mass-to-charge ratios; min, minutes.

The extraction yield, TPC, TFC, and the antioxidant activity of the cat's claw extract are shown in Table 1. The UHPLC/MS analysis revealed the presence of four pentacyclic (uncarine isomers) and three tetracyclic (rhynchophylline and corynoxeines) oxindole alkaloids. Although not quantified, uncarines appeared to be the most abundant ones (Figure 2). Other components detected in the extract were tentatively identified as 7-deoxyloganic acid [Mass 378.1764 m/z; retention time (RT) 15.08 min]; cincholic acid derivatives (Mass 812.4796 m/z; RT 21.20 and 22.68 min) and tomentoside (Mass 930.5426 m/z; RT 24.33 min).

**Table 1:** Characterization of *Uncaria tomentosa* extract.

			**Antioxidant activity**			
**Extraction yield**	**TPC**	**TFC**	**ABTS decolorization assay**	**DPPH radical scavenging assay**		**ORAC**
**(%)**	**(mg GAE/g dry extract)**	**(**μ**g QE/g dry extract)**	**(mmol TE/g dry extract)**	**(IC**_50_ μ**g/mL)**	**(mg TE/g dry extract)**	**(mmol TE/g dry extract)**
3.26 ± 0.27	240.69 ± 4.66	53.22 ± 1.46	3.57 ± 0.45	18.82 ± 0.38	318.41 ± 6.46	6.35 ± 0.37

TPC, total phenolic content; TFC, total flavonoid content; ABTS, 2,2′-azino-bis-(3-ethylbenzothiazoline-6-sulfonic acid); DPPH, 1,1-Diphenyl-2-picryl-hydrazyl; ORAC, oxygen radical absorbance capacity; GAE, gallic acid equivalents; QE, quercetin equivalents; TE, Trolox equivalents; IC_50_, half-inhibitory concentration. The data are shown as mean ± standard deviation.

### 3.2 Experiment 1: sperm samples under oxidative stress

The results of motility and kinetics of sperm samples under oxidative stress are shown in Table 2. In semen samples under oxidative stress induced by H_2_O_2_, there were no significant differences (*p* > 0.05) in sperm motility and kinetics when UT treatments were compared with the Ctr-Ox. However, all treatments, including Ctr-Ox, showed significant differences (*p* < 0.05) compared to the Ctr, except for STR. Conversely, under oxidative stress induced by Fe^2+^/Asc, some kinetics parameters (i.e., VCL and ALH) of UT 0.025 treatment did not differ (*p* > 0.05) from Ctr, which showed significantly higher values of these parameters than Ctr-Ox (*p* = 0.010 and *p* = 0.038, respectively).

**Table 2:** Effect of *Uncaria tomentosa* on sperm motility and kinetics on semen samples under oxidative stress.

**Oxidative stress inducer**	**Treatment**	**TM (%)**	**PM (%)**	**VAP (μm/s)**	**VCL (μm/s)**	**VSL (μm/s)**	**ALH (μm)**	**BCF (Hz)**	**LIN (%)**	**STR (%)**
**H**_**2**_ **O**_**2**_	Ctr	78.91 ± 4.69^a^	75.35 ± 5.68^a^	71.41 ± 8.81^a^	99.47 ± 15.54^a^	66.55 ± 7.17^a^	3.98 ± 0.61^a^	18.35 ± 0.98^a^	68.85 ± 4.48^a^	93.02 ± 2.24^b^
	Ctr-Ox	64.18 ± 4.81^bc^	70.28 ± 4.19^a^	38.40 ± 8.23^b^	59.88 ± 11.25^b^	37.05 ± 8.00^b^	2.42 ± 0.55^b^	15.69 ± 1.21^b^	61.80 ± 3.74^b^	95.89 ± 0.54^a^
	UT 1.6	57.58 ± 11.18^c^	67.36 ± 11.59^a^	33.79 ± 4.87^b^	55.00 ± 8.28^b^	32.50 ± 4.71^b^	2.24 ± 0.37^b^	14.92 ± 0.62^b^	59.64 ± 1.98^b^	95.75 ± 0.49^a^
	UT 0.4	67.49 ± 8.91^b^	74.52 ± 8.36^a^	39.54 ± 9.25^b^	60.69 ± 12.10^b^	38.04 ± 8.99^b^	2.57 ± 0.65^b^	15.05 ± 0.96^b^	60.47 ± 3.75^b^	95.62 ± 0.46^a^
	UT 0.1	66.64 ± 6.73^b^	64.17 ± 17.36^b^	38.89 ± 8.03^b^	62.92 ± 11.05^b^	37.43 ± 7.76^b^	2.52 ± 0.56^b^	15.34 ± 0.78^b^	61.60 ± 2.97^b^	95.75 ± 0.48^a^
	UT 0.025	63.76 ± 8.92^bc^	70.59 ± 7.50^a^	40.08 ± 10.45^b^	62.78 ± 14.79^b^	38.69 ± 10.14^b^	2.56 ± 0.70^b^	15.73 ± 0.91^b^	61.95 ± 1.98^b^	95.87 ± 0.59^a^
**Fe** ^**2+**^ **/Ascorbate**	Ctr	66.34 ± 20.27^a^	71.47 ± 8.38	59.57 ± 32.05^a^	85.55 ± 45.56^a^	56.38 ± 30.74^a^	3.34 ± 1.75^a^	16.80 ± 4.45^b^	60.43 ± 22.33^b^	86.83 ± 18.84^b^
	Ctr-Ox	44.67 ± 12.55^b^	64.65 ± 10.29	42.13 ± 12.51^a^	51.66 ± 17.12^b^	41.32 ± 12.20^ab^	2.30 ± 0.68^b^	20.06 ± 0.91^a^	82.72 ± 2.00^a^	98.01 ± 0.29^a^
	UT 1.6	43.43 ± 11.22^b^	64.7 ± 7.31	37.99 ± 11.22^b^	46.63 ± 14.84^b^	37.25 ± 10.94^b^	2.06 ± 0.59^b^	19.98 ± 0.93^a^	81.70 ± 1.43^a^	97.86 ± 0.27^a^
	UT 0.4	45.49 ± 9.85^b^	65.04 ± 7.66	41.82 ± 7.20^a^	52.44 ± 10.68^b^	40.93 ± 7.01^ab^	2.33 ± 0.42^b^	19.26 ± 1.06^a^	80.43 ± 2.40^a^	97.53 ± 0.32^a^
	UT 0.1	46.41 ± 12.02^b^	64.70 ± 9.09	42.00 ± 9.52^a^	51.84 ± 12.37^b^	41.02 ± 9.25^ab^	2.29 ± 0.48^b^	19.90 ± 0.90^a^	81.22 ± 1.25^a^	97.65 ± 0.17^a^
	UT 0.025	47.94 ± 11.29^b^	63.93 ± 6.45	49.27 ± 19.63^a^	62.82 ± 28.69^ab^	47.99 ± 18.62^ab^	2.72 ± 1.02^ab^	19.13 ± 1.37^a^	80.02 ± 4.74^a^	97.55 ± 0.95^a^

Different superscripts indicate significant differences (*p* < 0.05) between treatments within each ROS-generating system. Treatments: Ctr, control; Ctr-Ox, control with induced oxidative stress; UT 1.6, *Uncaria tomentosa* 1.6 μg/mL; UT 0.4, *Uncaria tomentosa* 0.4 μg/mL; UT 0.1, *Uncaria tomentosa* 0.1 μg/mL; UT 0.025, *Uncaria tomentosa* 0.025 μg/mL. TM, total motility; PM, progressive motility; VAP, average path velocity; VCL, curvilinear velocity; VSL, straight-line velocity; ALH, amplitude of lateral head displacement; BCF, beat-cross frequency; LIN, linearity; STR, straightness. The data are shown as the mean ± standard deviation of six replicates.

In semen samples under oxidative stress induced by H_2_O_2_ there were not significant differences on the plasma membrane integrity between any treatments (*p* > 0.05, Figure 1A). The induction of oxidative stress by Fe^2+^/Asc promoted a decrease in the percentage of sperm with an intact plasma membrane in the Ctr-Ox in comparison to the Ctr group (*p* < 0.01, Figure 1B). Interestingly, the percentage of sperm cells with intact membrane in the treatments UT 0.4, UT 0.1, and UT 0.025 was significantly higher than in the Ctr-Ox (*p* = 0.047, *p* = 0.009, and *p* = 0.008, respectively) and did not differ from the Ctr group (*p* > 0.05).

### 3.3 Experiment 2: sperm samples stored at 17°C

The results of sperm motility, kinetics, and plasma membrane integrity during the storage at 17°C are shown in Table 3 and Figure 1C. We found that there were not significant differences (*p* > 0.05) between the Ctr and the samples supplemented with cat's claw extract in any sperm parameters. Albeit not significant (*p* > 0.05), at 48 h and 96 h, some treatments showed higher means of TM and some kinetic parameters than the Ctr. For example, at 48 h, UT 0.4 and UT 0.1 showed higher TM, VAP, VCL and ALH, when compared to the Ctr; at 96 h, all the treatments showed higher VAP and VSL than the Ctr.

**Table 3:** Effects of *Uncaria tomentosa* on sperm motility and kinetics during semen storage at 17°C.

**Time**	**Treatment**	**TM (%)**	**PM (%)**	**VAP (μm/s)**	**VCL (μm/s)**	**VSL (μm/s)**	**ALH (μm)**	**BCF (Hz)**	**LIN (%)**	**STR (%)**
**48 h**	Ctr	71.99 ± 4.56	52.04 ± 8.83	86.16 ± 9.34	149.04 ± 17.42	69.05 ± 7.98	5.14 ± 0.48	15.62 ± 0.57	47.67 ± 4.43	79.39 ± 5.11
	UT 1.6	71.99 ± 6.68	49.97 ± 9.43	83.87 ± 7.22	144.63 ± 11.43	66.29 ± 10.00	4.99 ± 0.43	16.01 ± 1.09	47.55 ± 6.36	78.35 ± 5.59
	UT 0.4	74.35 ± 7.00	49.65 ± 11.97	86.82 ± 9.52	149.37 ± 12.11	68.20 ± 13.44	5.21 ± 0.45	15.79 ± 1.01	46.55 ± 7.52	77.45 ± 8.37
	UT 0.1	77.68 ± 4.17	49.96 ± 13.19	86.39 ± 6.07	151.54 ± 9.19	66.56 ± 11.89	5.19 ± 0.27	15.53 ± 1.50	45.58 ± 9.70	76.33 ± 10.16
	UT 0.025	71.56 ± 7.39	44.88 ± 12.85	84.17 ± 8.69	152.04 ± 16.16	61.49 ± 10.66	5.13 ± 0.53	15.00 ± 1.06	42.34 ± 7.72	72.67 ± 8.72
**96 h**	Ctr	64.09 ± 10.08	46.97 ± 8.08	82.29 ± 9.75	139.38 ± 11.19	65.85 ± 9.91	5.08 ± 0.32	15.89 ± 1.45	49.48 ± 6.42	79.46 ± 4.07
	UT 1.6	62.67 ± 8.66	51.13 ± 9.58	85.31 ± 7.73	137.21 ± 20.70	71.50 ± 8.09	5.01 ± 0.51	16.62 ± 1.35	55.49 ± 10.46	83.14 ± 6.68
	UT 0.4	63.19 ± 6.56	48.54 ± 8.49	88.38 ± 6.47	146.39 ± 18.69	72.26 ± 4.11	5.29 ± 0.51	16.34 ± 0.73	52.43 ± 7.52	81.34 ± 6.12
	UT 0.1	62.32 ± 5.02	46.72 ± 11.72	87.57 ± 8.36	149.02 ± 23.19	69.47 ± 9.56	5.32 ± 0.58	15.81 ± 1.29	49.97 ± 11.04	78.76 ± 10.52
	UT 0.025	64.93 ± 4.82	48.26 ± 11.11	84.39 ± 7.55	145.32 ± 24.12	65.95 ± 7.00	5.12 ± 0.56	15.95 ± 1.03	48.87 ± 11.72	77.68 ± 11.93

Different superscripts indicate significant differences (*p* < 0.05) between treatments within each given time. Treatments: Ctr, control; UT 1.6, *Uncaria tomentosa* 1.6 μg/mL; UT 0.4, *Uncaria tomentosa* 0.4 μg/mL; UT 0.1, *Uncaria tomentosa* 0.1 μg/mL; UT 0.025, *Uncaria tomentosa* 0.025 μg/mL. TM, total motility; PM, progressive motility; VAP, average path velocity; VCL, curvilinear velocity; VSL, straight-line velocity; ALH, amplitude of lateral head displacement; BFC, beat-cross frequency; LIN, linearity; STR, straightness. The data are shown as the mean ± standard deviation of six replicates.

Moreover, the percentage of sperm with intact membrane in UT treatments did not differ significantly (*p* > 0.05) from the Ctr. Nevertheless, the average of the sperm with intact membrane of the UT treatments was higher than the Ctr (*p* > 0.05).

### 3.4 Antimicrobial assays

#### 3.4.1 Contaminating bacteria isolated from pure semen

The contaminating bacteria isolated from pure semen are shown in Table 4. A total of 15 bacterial species were isolated including five Gram+ and 10 Gram– species. The Gram– bacteria prevailed in number of species as well as in occurrence frequency, whereas *C. koseri, E. faecalis, K. aerogenes, P. aeruginosa*, and *S. maltophilia* were the most frequently isolated.

**Table 4:** Contaminating bacteria isolated from pure semen.

**Species**	**Gram classification**	**Frequency per sampling^a^**	
		**n**	**%**
*Bacillus cereus*	G+	1	3
*Bacillus simplex*	G+	1	3
*Enterococcus faecalis*	G+	18	47
*Enterococcus faecium*	G+	1	3
*Staphylococcus hominis*	G+	1	3
*Citrobacter koseri*	G–	20	53
*Escherichia coli*	G–	7	18
*Klebsiella aerogenes*	G–	11	29
*Klebsiella pneumoniae*	G–	2	5
*Leclercia adecarboxylata*	G–	1	3
*Morganella morganii*	G–	1	3
*Proteus mirabilis*	G–	3	8
*Pseudomonas aeruginosa*	G–	11	29
*Serratia liquefaciens*	G–	2	5
*Stenotrophomonas maltophilia*	G–	10	26

a total number of samplings = 38; G+, Gram-positive; G–, Gram-negative.

#### 3.4.2 MIC and bacterial kinetic growth

The individual MICs of *U. tomentosa* extract and gentamicin for all the bacteria tested are summarized in Table 5. In general, at the concentrations tested, UT extract was more effective against Gram+ bacteria, inhibiting all the strains tested. Nevertheless, the extract was active also against several Gram– semen isolates (i.e., *C. koseri, P. aeruginosa*, and *S. maltophilia*).

**Table 5:** Minimum inhibitory concentration of *Uncaria tomentosa* and gentamicin on commercial bacteria and bacteria isolated from porcine semen.

**Species**	**Gram classification**	**Strain**	**MIC (**μ**g/mL)**	
			***Uncaria tomentosa***	**Gentamicin**
*Enterococcus faecalis*	G+	ATCC 29212	256–512	32
*Enterococcus faecalis*	G+	IS 21751	128	16
*Staphylococcus aureus*	G+	ATCC 29213	1,024	2
*Staphylococcus epidermidis*	G+	IS 2152	128	32
*Staphylococcus pasteuri*	G+	IS 22062	1,024	1
*Citrobacter koseri*	G−	IS 2111	1,024	2
*Escherichia coli*	G−	ATCC 25922	>1,024	2
*Escherichia coli*	G−	IS 21710	>1,024	8
*Klebsiella aerogenes*	G−	IS 2137	>1,024	4
*Pseudomonas aeruginosa*	G−	ATCC 27853	≥1,024	2
*Pseudomonas aeruginosa*	G−	IS 2216	256–512	2
*Stenotrophomonas maltophilia*	G−	IS 2107	512–1,024	1

G+, Gram-positive; G−, Gram-negative; IS, Isolated from Semen (strains isolated from porcine semen); ATCC, American Type Culture Collection (commercial strains); MIC, minimum inhibitory concentration.

**Figure 3:**
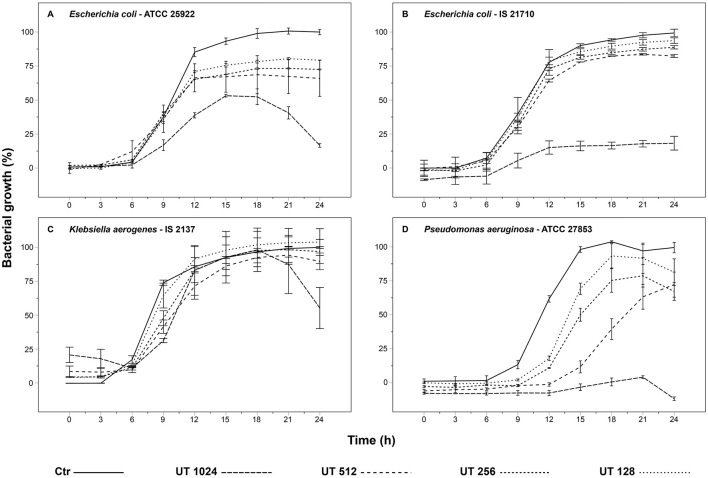
Effect of *Uncaria tomentosa* extract on bacterial growth: **(A)** *Escherichia coli* (ATCC 25922); **(B)** *Escherichia coli* (IS 21710); **(C)** *Klebsiella aerogenes* (IS 2137); **(D)** *Pseudomonas aeruginosa* (ATCC 27853). IS, Isolated from Semen (strains isolated from porcine semen); ATCC, American Type Culture Collection (commercial strains). Treatments: Ctr, control; UT 1,024, *Uncaria tomentosa* 1,024 μg/mL; UT 512, *Uncaria tomentosa* 512 μg/mL; UT 256, *Uncaria tomentosa* 256 μg/mL; UT 128, *Uncaria tomentosa* 128 μg/mL. The data are shown as the mean ± standard deviation of two replicates.

The growth kinetics of the bacteria, which showed a MIC ≥ 1,024 μg/mL, are shown in Figure 3. Although inhibition of bacterial growth may not have been detected by the unaided eye, all cultures showed a decrease or delay in growth over 24 h at least at the highest concentration of the UT extract. The peak of bacterial growth of the commercial strain *E. coli* (ATCC 25922) was reduced by about half by UT 1,024 treatment. Additionally, the stationary phase was brief, and only at this concentration it was possible to observe the death phase after 24 h. The remaining curves (UT 512, UT 256, and UT 128) closely resembled the control group, exhibiting only a moderate reduction in bacterial growth ranging from 20 to 30% (Figure 3A). The bacterial growth of *E. coli* isolated from the boar semen (IS 21710) was reduced by about 80% from the treatment UT 1,024. The other tested concentrations (i.e., UT 512, UT 256, and UT 128) exhibited a slight decrease in bacterial growth (Figure 3B). Regarding the growth of *K. aerogenes* isolated from the semen samples (IS 2137), across all concentrations, the exponential phase showed a slight delay compared to the control. The UT 1,024 treatment reached the same bacterial growth as the control between 12 and 18 h. However, the final 6 h of incubation revealed the initiation of a death phase, resulting in a reduction of growth by half. The remaining concentrations (i.e., UT 512, UT 256, and UT 128) showed minimal difference from the control (Figure 3C). Against the commercial strain *P. aeruginosa* (ATCC 27853), all treatments exhibited a delay in the exponential phase compared to the control, with lower concentrations (UT 256 and UT 128) showing a 3-h delay and higher concentrations (UT 1,024 and UT 512) a 6-h delay. The UT 1,024 treatment demonstrated near-complete growth inhibition, while the other treatments reduced growth by 10–30% (Figure 3D).

## 4 Discussion

In this study, cat's claw extract was tested for the first time on sperm cells and against bacteria isolated from semen. This plant protected the sperm membrane under oxidative stress and showed antibacterial properties against Gram+ and Gram– bacteria. The treatments ranging from 0.4 to 0.025 μg/mL protected the integrity of the sperm membrane and preserved some key sperm kinetic parameters (i.e., VCL and ALH) in samples under oxidative stress (Fe^2+^/Asc). The *U. tomentosa* extract, at all tested concentrations, was not toxic to sperm cells up to 96 h of storage at 17°C as it keeps sperm parameters unaltered or even showed higher values when compared to the Ctr group. All tested Gram+ bacteria exhibited MICs within a range from 1,024 to 128 μg/mL. In contrast, MICs for Gram– bacteria varied more widely. Some Gram– bacteria isolated from the semen (*C. koseri, P. aeruginosa*, and *S. maltophilia*) showed complete growth inhibition with MICs from 1,024 to 256 μg/mL; it is known that the presence of those bacteria damages the sperm cells and decreases the quality of the semen (33, 36, 37). Albeit without complete inhibition, the remaining Gram– strains showed a decrease in growth between 90 and 50% compared to the control. The use of *U. tomentosa* during sperm storage and under oxidative stress has shown promising results due to the antioxidant and antimicrobial properties of the extract.

In the present study, the ethanolic extract of *U. tomentosa* bark showed a lower yield and TFC when compared to similar studies (12, 38–40), while the TPC was within the range previously reported [554.6–196.2 mg/g; (38, 41, 42)]. However, it is important to note that the extraction yield and secondary metabolites content can vary depending on the variety of plant used, extraction method, solvent, and extraction time. Our data consistently supported the previously documented antioxidant potential of the extract (10). The ABTS decolorization assay yielded a value comparable to that reported by Júnior et al. (41) but significantly exceeding that found by Pilarski et al. (42). The IC_50_ for DPPH radical scavenging was found to be slightly lower than the one reported by Sandoval et al. (43). Finally, ORAC assay results indicated an antioxidant capacity consistent with values reported for bark ethanolic extracts by Navarro-Hoyos et al. (44). Our UHPLC/MS analysis of the extract revealed the presence of alkaloids, a major compound group in the *Uncaria* genus (45), and terpenes. Specifically, we identified oxindole alkaloids, including both pentacyclic and tetracyclic variants. Among pentacyclic oxindole alkaloids, four uncarine isomers were detected, while corynoxeine and rhynchophylline were identified as tetracyclic oxindole alkaloids. These compounds have been previously reported in this plant (5, 46–48). Due to the lack of standards, distinguishing isomers within each molecule was not feasible. However, by examining peak intensities and counts in the chromatogram, we estimated that uncarine alkaloids were the most abundant in our extract, followed by rhynchophylline and corynoxeine. Based on the observed alkaloid profile, we conclude that the cat's claw bark employed in this experiment is from the pentacyclic alkaloid type (4, 5). Previous studies demonstrated the antibacterial and/or antioxidant activity of all the alkaloids mentioned. Uncarine and rhynchophylline exhibited antibacterial activity against both Gram+ and Gram– bacteria (6, 49, 50), while rhynchophylline and corynoxeine have previously been reported to possess antioxidant activity (51, 52). Among the terpenes, we identified the monoterpene glycoside 7-deoxyloganic acid, two glycosylated derivatives of the pentacyclic triterpene cincholic acid, and the tomentoside, a potential pyroquinovic or pyrocincholic acid derivate. The literature reports the occurrence of these compounds in *U. tomentosa* (53–55).

Nowadays, extracts from plants and biomolecules isolated from natural sources are starting to be added to extenders to improve semen preservation, as they enhance sperm quality through their antioxidant and antimicrobial properties (13, 32, 56). Our study demonstrates the antioxidant properties of UT ethanolic extract on sperm cells when incorporated into the BTS extender. The protective effect of *U. tomentosa* observed in samples under Fe^2+^/Asc but not under H_2_O_2_ might be due to the fact that only the former ROS-generating system was able to induce a detrimental effect on the sperm membrane. Moreover, the percentage of spermatozoa with intact plasma membrane was similar between Ctr and Ctr-Ox groups in samples exposed to H_2_O_2_ in agreement with our previous findings (30). The different ROS produced by the two inducers might explain this phenomenon. The main product of the oxidative cascade initiated by Fe^2+^/Asc inducer is the hydroxyl radical (•OH) produced by the ascorbate-driven Fenton reaction (57); while, H_2_O_2_ can directly damage sperm cells and promote the production of other ROS (58, 59). Moreover, a key difference between these ROS is that while H_2_O_2_ can be neutralized by several antioxidant enzymes like catalase, glutathione peroxidase, and peroxiredoxins, no known enzymatic system eliminates the •OH, likely due to its high reactivity (60). Both •OH and H_2_O_2_ are ROS, but only the former is a free radical. In our research, among the samples under oxidative stress induced by Fe^2+^/Asc, the treatment UT 0.025 preserved the kinetic parameters VCL and ALH, with no significant difference compared to the Ctr group. The importance of these kinetic parameters for swine reproduction efficiency is well established. Broekhuijse et al. (61) demonstrated that VCL and ALH, along with other parameters, influence the farrowing rate and litter size in the domestic pig. Similarly, a recent study by Fernández-López et al. (62) confirmed that greater values of kinetic parameters, including VCL and ALH, are associated with reproductive success in boars. Under the same conditions, the UT treatments ranging from 0.4 to 0.025 μg/mL significantly enhanced sperm plasma membrane integrity compared to the Ctr-Ox. This finding is in agreement with previous research by Duchnowicz et al. (63), who reported that secondary metabolites present in *U. tomentosa* extract interact with the plasma membrane of human erythrocytes, shielding them from oxidative damage. Karonen (64) described that phenolic compounds can interact with lipid bilayers in the cell membrane, thanks to the interaction influenced by the specific chemical properties of both the membrane and the phenols. Notably, secondary metabolites offer additional benefits; phenols and terpenes act as electron and H-atom donors to scavenge ROS (65, 66), while phenols and alkaloids can chelate metals such as iron (67–69). Based on these combined mechanisms, we can postulate that the cat's claw extract used in our study preserved membrane integrity through two main pathways: directly interacting with membrane lipids and indirectly neutralizing products generated by the ascorbate-driven Fenton reaction. Further analyses (e.g., lipid peroxidation, ROS levels and, superoxide dismutase and catalase activity) are needed to deepen the mechanism of antioxidant activity of this plant extract.

The *U. tomentosa* extract demonstrated antibacterial activity in addition to its antioxidant properties. We evaluated its efficacy against a panel of five Gram+ and seven Gram– bacteria, including strains isolated from pure and diluted semen, at concentrations ranging from 1,024 to 8 μg/mL. Most of the bacteria identified by MALDI-TOF technique in pure boar semen were already identified by previous studies (70–73), with the exception of *Leclercia adecarboxylata* and *Staphylococcus hominis*, whose origin could be attributed to the pig feed and skin, respectively (74, 75).

Previous studies have documented the antimicrobial potential of *U. tomentosa* extracts, preparations, and isolated compounds against both Gram+ (6, 11, 76) and Gram– bacteria (50, 76). Our findings confirm previous studies highlighting the antibacterial efficacy of *U. tomentosa* extract, particularly against Gram+ strains. These bacteria were susceptible to UT extract at concentrations ranging from 1,024 to 128 μg/mL. The UT extract showed inhibition at concentrations between 1,024 and 256 μg/mL against the Gram– bacteria *C. koseri, P. aeruginosa*, and *S. maltophilia*, isolated from semen. In other studies, it was demonstrated that those bacteria can negatively affect sperm quality. Sone (33) showed that *Citrobacter* sp. causes a decrease in pH, sperm motility, and acrosome integrity. Likewise, *P. aeruginosa* decreases the percentages of total and progressive sperm motility, sperm viability, and acrosome integrity (37). Finally, Althouse et al. (36) reported that *S. maltophilia*, frequently isolated from porcine semen, exhibits spermicidal activity. The remaining Gram– bacteria (the isolates from semen *E. coli* and *K. arerogenes* and the commercial strains *E. coli* and *P. aeruginosa*) only showed a decrease in bacterial growth, although the extract did not completely inhibit it. Interestingly, our results align with those of Kloucek et al. (12), who reported similar MIC values for ethanolic extract of *U. tomentosa* bark against different bacterial strains. Specifically, MICs for *E. faecalis* (ATCC 29212) and two strains of *S. aureus* (ATCC 25923, ATCC 29213) showed values similar to our findings. Neither *E. coli* (ATCC 25922) nor *P. aeruginosa* (ATCC 27853) exhibited sensitivity to the UT ethanolic extract at the concentration of 1,024 μg/mL in either study. Furthermore, the UT extract demonstrated greater activity against strains isolated from semen than against commercially obtained strains of the same species or genera. The antibacterial activity is attributed to the secondary metabolites present in the extract. The phenols prevent bacterial growth by destroying bacteria's cell walls and membranes, inhibiting enzyme activities, and interfering with biofilm formation (77). Terpenes, due to their lipophilic nature, can penetrate microbial cell walls (78) and subsequently damage the bacterial membrane, leading to altered permeability and the release of cellular constituents (79). Finally, C3-substituted oxindoles can have antimicrobial activity (80). Since all oxindole alkaloids detected in our study are C3-substituted, they may contribute to the observed antibacterial effects.

In the present study, the UT extract concentrations that showed antibacterial activity were higher than those tested on boar sperm cells. Thus, it would be appropriate to adjust the concentrations in order to maintain the antimicrobial activity and minimize the toxicity against sperm cells. Moraes et al. (81) demonstrated that UT extract can enhance cell damage of *Candida* ssp. when combined with common antifungals, suggesting a synergistic or additive effect. We must consider that the current antimicrobial assays were performed in a standard cultivation medium. We can thus assume that the antimicrobial effect of the UT extract can be enhanced synergistically in combination with common components of sperm extenders, as has previously been reported in the case of ethylenediaminetetraacetic acid combined with natural compounds (32, 82).

In conclusion, *U. tomentosa* showed both cytoprotective effects and antibacterial properties when tested on boar sperm cells and against porcine semen-isolated bacteria. Our findings revealed its protective effect on sperm cell membrane against oxidative stress induced by Fe^2+^/Asc at concentrations ranging from 0.4 to 0.025 μg/mL. Additionally, the extract inhibited the growth of six out of eight semen-isolated bacteria tested at concentrations ranging from 1,024 to 128 μg/mL. Further studies are needed to optimize cat's claw extract as a semen additive. Careful concentration adjustments are required to balance antibacterial efficacy with sperm cell safety. As a future direction, investigating the combination of *U. tomentosa* extract with other botanical extracts, isolated natural compounds, or conventional antibiotics could be explored to maintain antimicrobial and antioxidant benefits while minimizing cytotoxicity.

## Data Availability

The original contributions presented in the study are included in the article/Supplementary material, further inquiries can be directed to the corresponding authors.
